# Comprehensive care for hereditary angioedema: lessons learned from HAEmophilia Treatment Centers

**DOI:** 10.1186/s13223-026-01042-0

**Published:** 2026-05-13

**Authors:** Kelsey Uminski, Dawn Goodyear

**Affiliations:** 1 Division of Hematology and Hematological Malignancies, Department of Medicine, Foothills Medical Centre, University of Calgary, 1403 29 St NW, Calgary, AB T2N 2T9 Canada; 2 Southern Alberta Rare Blood and Bleeding Disorders Comprehensive Care Program, Calgary, AB Canada

**Keywords:** Hereditary angioedema, Hemophilia, Comprehensive care, Rare diseases, Emergency preparedness, Patient education, Care coordination

## Abstract

Hereditary angioedema (HAE) is a rare, chronic disorder that requires coordinated, multidisciplinary care extending beyond acute attack management. Drawing on the successful comprehensive care framework established in Hemophilia Treatment Centers, this manuscript proposes a structured model for HAE care that emphasizes centralized care coordination, multidisciplinary teams, emergency preparedness, patient and caregiver education, psychosocial support, equitable access to emerging therapies, and integrated research and advocacy. By adapting lessons learned from hemophilia care, the proposed model aims to improve patient outcomes, empower individuals living with HAE, and provide a roadmap for implementing standardized, patient-centered care in rare disease management.

To the Editor,

Hereditary angioedema (HAE) and hemophilia are rare disorders, requiring specialized and coordinated care. There are numerous publications detailing the necessity of ‘comprehensive’ acute care plans for HAE, largely focused on emergent management of HAE attacks in the acute care setting such as emergency rooms [1–3]. However, the need for truly comprehensive, multidisciplinary care coordination, recognizing the complexities of HAE as a chronic disease, with care needs extending beyond the acute care setting is gaining increased recognition [4]. The International/Canadian Hereditary Angioedema Guideline emphasizes that optimal HAE care requires an individualized treatment approach, including both on-demand and long-term prophylactic therapy, patient education, and shared decision-making to improve disease control and quality of life [4, 5]. Additionally, guidelines advocate for specialized HAE centers to provide multidisciplinary care, integrating emergency care providers, allergists, immunologists, hematologists, and other specialists to ensure comprehensive disease management [4, 5]. Notably, these recommendations have been consistently emphasized across multiple iterations of international guidelines over time, and have also been proposed in earlier literature [6], yet implementation of comprehensive, multidisciplinary care models remains variable, highlighting a persistent gap in care delivery. The evolving treatment landscape further reinforces the importance of coordinated care, given the expanding array of therapeutic options, including novel prophylactic agents and emerging gene-based therapies [5, 7, 8]. While the care of HAE has largely fallen under the scope of allergists and immunologists, or hematologists in some Canadian regions, the complexity and impact of the condition necessitates a multidisciplinary approach similar to that utilized in hemophilia care.

Hemophilia is an inherited bleeding disorder characterized by deficiencies in clotting factors VIII or IX (hemophilia A or B, respectively), leading to recurrent bleeding events, most often musculoskeletal [9]. The consequences of such bleeding events, is hemophilic arthropathy, and disability over time [9]. Historically, hemophilia management was limited to the management of acute bleeding events, leading to hospitalization, morbidity and reduced life expectancy [9]. The establishment of Hemophilia Treatment Centers (HTCs) in the 1960s marked a paradigm shift in the delivery of care for individuals with hemophilia [10]. These centers introduced a multidisciplinary approach, integrating hematologists, nurses, physiotherapists, psychologists, and social workers to provide holistic and preventive care [9, 10]. Over the years, the HTC model of care has evolved to prioritize education, patient self-management at home, and prophylactic treatments, enabling patients to lead more independent lives, and additional team members including dentists, orthopedic surgeons and obstetricians and gynecologists. [9, 10]

Hemophilia management has seen a large expansion of therapeutic options in recent years [11], with gene therapy further expanding this evolution, correcting the underlying genetic defect by delivering functional copies of the deficient clotting factor gene [12]. Gene therapy has emerged as a transformative therapeutic approach in hemophilia, with two adeno-associated virus vector-based gene therapies-valoctocogene roxaparvovec [13, 14] (Roctavian, BioMarin) and etranacogene dezaparvovec [15] (Hemgenix, CSL Behring)—now commercially available. HTCs have adapted to these advancements in therapies by integrating comprehensive gene therapy readiness programs, which include patient eligibility assessments, biosafety considerations, and structured long-term monitoring protocols, and both patient and health care provider education on emerging treatments [16, 17]. A similar therapeutic expansion is currently being observed in the management of HAE, requiring preparation on the part of HAE providers to ensure individualized treatment [5, 7]. This manuscript explores how the comprehensive care principles established within HTCs for hemophilia can be adapted and applied to HAE, a similarly rare and complex disorder. By leveraging lessons learned from hemophilia care, we aim to propose a model that addresses the unique challenges of HAE management while improving patient outcomes and quality of life.

## Comprehensive care model in hemophilia

Treatment for bleeding events in hemophilia often involves intravenous (IV) administration of clotting factor concentrates, a practice that parallels the use of IV C1-esterase inhibitor therapy for acute attacks in HAE. In both conditions, the ability to self-administer IV therapies is a cornerstone of effective disease management. Nursing staff in comprehensive care clinics play a critical role in supporting patients and their families in developing the necessary IV skills, fostering independence, and improving access to timely treatment. This approach reduces the need for hospital-based care and empowers patients to manage their conditions more effectively at home.

The development of HTCs was a pivotal advancement in hemophilia care, significantly reducing morbidity and mortality associated with the condition [10]. Historically, individuals with severe hemophilia faced a high risk of early death due to uncontrolled bleeding, joint destruction, and limited access to effective treatments. The HTC model was introduced to address these challenges, creating a framework that integrates multidisciplinary care. Hematologists, nurses, physiotherapists, social workers, psychologists, dentists, orthopedic surgeons, and obstetrician-gynecologists collaborate within HTCs to provide holistic care, significantly improving patient outcomes [9]. Data from longitudinal studies have demonstrated that comprehensive care provided through HTCs leads to reduced hospitalization rates, improved joint health, and increased life expectancy for individuals with hemophilia [9, 10].

A fundamental component of comprehensive care is emergency preparedness, ensuring that patients have individualized care plans that outline recommended hemostatic therapy during bleeding episodes. These plans are documented in both the patient’s medical record and on a personal wallet card, allowing for timely interventions during emergencies. Patient education is central to the HTC model, empowering individuals to recognize and manage bleeding episodes through structured self-management programs. This education has played a crucial role in shifting hemophilia care away from hospitals and toward home-based treatment, dramatically improving quality of life [10].

The HTC model also ensures 24/7 access to hematology specialists, providing after-hours support that reduces reliance on emergency departments and mitigates delays in receiving appropriate care. Additionally, HTCs play a vital role in perioperative and peripartum planning, tailoring hemostatic management to minimize surgical and obstetrical bleeding risks. During hospital admissions, HTC teams provide direct support, ensuring continuity of care and expert oversight in managing complex cases.

As treatment options evolve, HTCs have adapted to meet emerging challenges, including the preparation for gene therapy. With gene therapy now a viable treatment option, HTCs are increasingly involved in patient eligibility assessments, biosafety considerations, and long-term follow-up to optimize outcomes [17, 18]. Data registries remain an integral part of comprehensive care, supporting research, quality improvement, and individualized treatment planning by tracking outcomes and identifying areas for innovation [16]. These collective advancements highlight the substantial improvements in morbidity and mortality following the establishment of comprehensive care programs, underscoring the importance of a structured, multidisciplinary approach in optimizing patient outcomes in hemophilia.

## Proposed framework for HAE comprehensive care centers

The development of a structured, multidisciplinary model for HAE care has the potential to significantly improve patient outcomes, much like the impact of HTCs in bleeding disorders. The World Federation of Hemophilia benchmarks for comprehensive hemophilia care, internationally adopted to standardize treatment and enhance access to life-saving therapies, were developed with significant contributions from Canadian clinicians and researchers. A similar gold standard for HAE care could be developed within Canada, leveraging existing expertise in rare disease management and serving as a model for international adoption. By implementing a structured framework, Canadian HAE treaters have an opportunity to create a globally recognized, standardized approach that ensures equitable access to comprehensive care, regardless of geographic location or healthcare system differences.

**Fig. 1:**
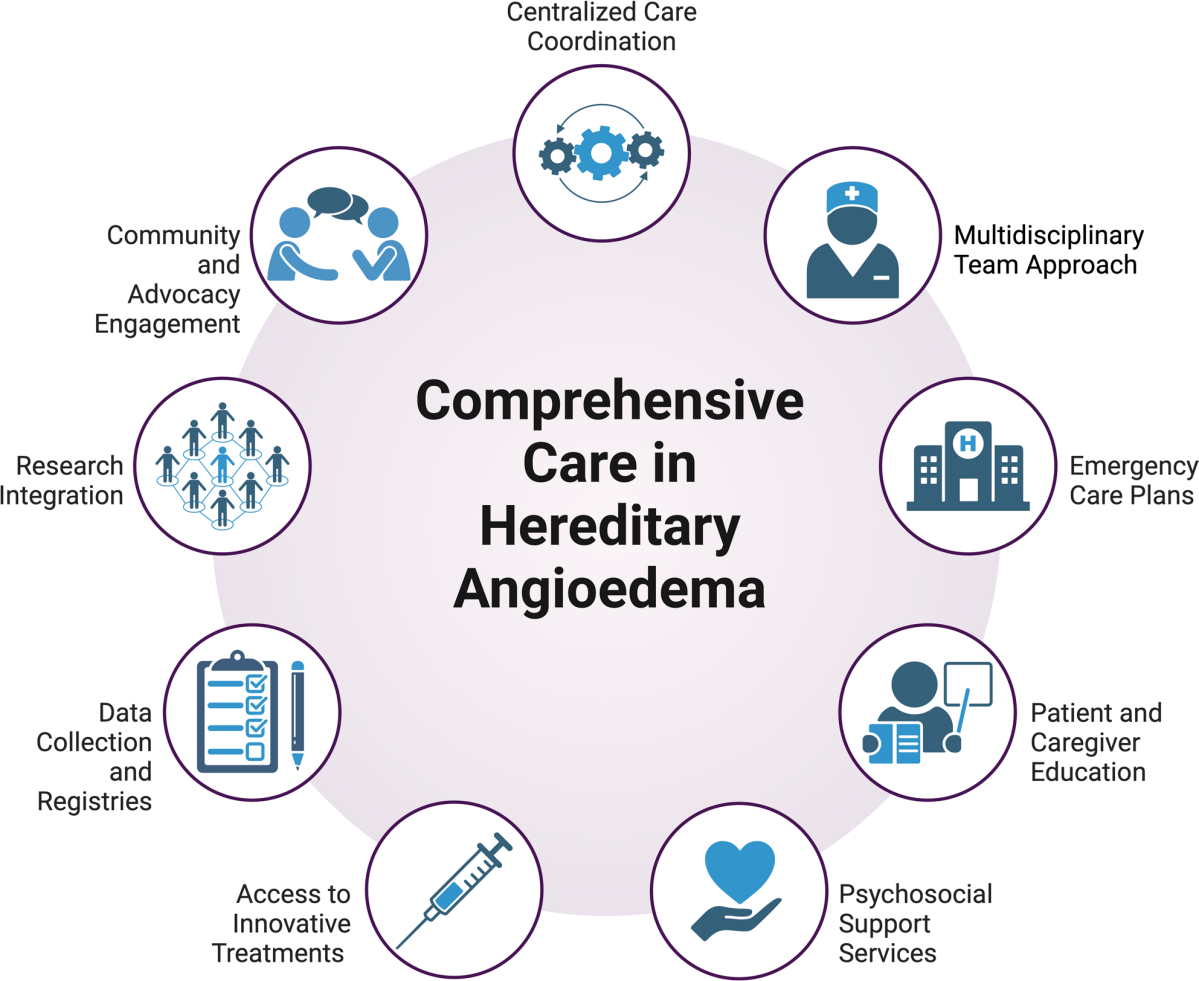
Model for comprehensive care in hereditary angioedema. Created with BioRender (https://www.biorender.com)

The framework in Fig. 1 proposes nine essential components to a comprehensive care model for HAE based on lessons learned from HTCs. These components include:

1. **Centralized Care Coordination**:
  Designate HAE comprehensive care centers as hubs for diagnosis, treatment planning, and ongoing management.
  Designate a care coordinator to streamline communication between providers, support access to therapies and ensure continuity of care including coordinated perioperative and peri/postpartum management to mitigate the risk of HAE attacks triggered by surgical procedures, labor, and delivery.
  Establish structured transition pathways from pediatric to adult care, including patient education and coordination between pediatric and adult providers, to ensure continuity of care and minimize gaps during this high-risk period.
2. **Multidisciplinary Team Approach**:
  Incorporate allergists and immunologists, hematologists, or other physicians specialized in HAE care, nurses, psychologists, social workers, dentists, genetic counselors, and pediatric providers into the care team to address the full spectrum of patient needs across the lifespan.
3. **Emergency Care Plans**:
  Develop individualized emergency care plans for all HAE patients, outlining specific treatment protocols for the management of acute attacks, including specialist contact information.
  Train emergency department (ED) staff on the use of first-line therapies, such as C1-esterase inhibitor concentrates and bradykinin receptor antagonists.
  Educate ED staff on differentiating HAE from histamine-mediated angioedema, emphasizing the lack of response to epinephrine, antihistamines, and corticosteroids in HAE and the importance of early administration of targeted therapies.
4. **Patient and Caregiver Education**:
  Implement structured education programs on HAE pathophysiology, attack triggers, and self-administration of therapies.
  Provide tools, such as HAE attack cards, to empower patients in acute care settings.
  Ensure patients have access to on-demand therapies and educate them on bringing their prescribed acute treatment (e.g., C1-esterase inhibitor concentrates, bradykinin receptor antagonists) to ED to avoid treatment delays.
5. **Psychosocial Support Services**:
  Address the emotional and mental health challenges associated with HAE through psychosocial interventions, including counseling and support groups.
6. **Access to Innovative Treatments**:
  Ensure equitable access to advanced therapies.
  Advocate for coverage and affordability of cutting-edge treatments.
7. **Data Collection and Registries**:
  Establish a national HAE registry to track patient outcomes, treatment efficacy, care quality, and disease burden.
  Include patient-reported outcomes to assess quality of life, treatment satisfaction, and the psychosocial impact of HAE.
  Utilize registry data to guide quality improvement initiatives, optimize treatment strategies, and support ongoing research.
8. **Research Integration**:
  Promote participation in clinical trials to explore novel therapies and understand the natural history of HAE.
  Foster collaborations with international research networks to advance global knowledge of HAE.
9. **Community and Advocacy Engagement**:
  Partner with patient-led organizations to raise awareness, improve education, and advocate for policies that enhance access to care.
  Empower patients to become active participants in their care and broader advocacy efforts.
  Collaborate with the Canadian Hereditary Angioedema Network (CHAEN), which supports HAE care through advocacy, best practice development, and resource dissemination for both patients and providers, ensuring standardized and equitable access to care.

These components serve as the foundation for building a robust and effective comprehensive care model for HAE patients, ensuring that all aspects of their health and wellbeing are addressed.

## Importance of comprehensive care for research and advocacy

Comprehensive care centers serve not only to deliver high-quality clinical care but also serve as hubs for advancing research and rare disease advocacy. These centers facilitate participation in clinical trials, fostering innovation in treatment modalities. Registries maintained by comprehensive care programs enable the collection of longitudinal data, providing insights into disease progression, treatment outcomes, and quality of life. Furthermore, these programs empower patients to participate in advocacy efforts, raising awareness about rare diseases and influencing policy to improve access to care and funding for research. By integrating research and advocacy into the care model, comprehensive care centers amplify their impact beyond individual patient outcomes, contributing to the broader rare disease community.

## Lessons from HTC implementation

The HTC model has demonstrated the transformative impact of centralized, multidisciplinary care in managing rare and complex disorders. Despite longstanding recognition of the importance of comprehensive care in HAE, implementation has been inconsistent across centers. A key distinction between hemophilia and HAE care is the current distribution of providers. While hemophilia care is largely centralized within academic HTCs, HAE may be managed by community-based clinicians, which may present challenges to implementing fully integrated comprehensive care models. Contributing factors likely also include variability in access to multidisciplinary resources and institutional support. While a detailed exploration of implementation barriers is beyond the scope of this manuscript, these challenges underscore the need for coordinated care networks linking community providers with specialized centers, and highlight the role of this framework as an advocacy tool to support the development of comprehensive HAE care.

A key lesson from HTCs is the importance of equitable access to comprehensive care, ensuring that all patients—regardless of location—receive timely, evidence-based treatments. By standardizing protocols, facilitating coordinated care, and integrating supportive services such as physiotherapy, psychosocial support, and emergency preparedness, HTCs have improved long-term outcomes and quality of life for hemophilia patients. Additionally, data collection through patient registries has played a vital role in advancing research, guiding treatment decisions, and shaping health policy, further demonstrating the impact of an organized care model. HTCs have also emphasized structured transition programs from pediatric to adult care, a principle that is equally relevant in HAE to ensure continuity and prevent gaps in management during vulnerable periods.

Applying these principles to HAE care presents an opportunity to optimize disease management, improve patient outcomes, and enhance quality of life. Like hemophilia, HAE requires timely access to specialized therapies and individualized treatment plans to prevent life-threatening complications. Establishing HAE comprehensive care centers, modeled after HTCs, would centralize expertise, streamline emergency and long-term management, and provide patients with structured education and support. Moreover, the development of national HAE registries and research initiatives would contribute to improving treatment protocols, assessing patient-reported outcomes, and ensuring equitable access to novel therapies. By leveraging lessons learned from hemophilia care, we aim to propose a structured and actionable model, grounded in evidence from hemophilia comprehensive care, that addresses the unique challenges of HAE management while improving patient outcomes and quality of life.

## Conclusion

Comprehensive care has transformed the management of hemophilia, setting a precedent for rare disease care. We advocate for a similar paradigm shift in HAE management, with the establishment of comprehensive care centers that prioritize multidisciplinary collaboration, patient education, emergency preparedness, research, and advocacy. This model has the potential to reduce morbidity, enhance quality of life, and empower patients living with HAE. By building on the successes of the HTC framework, and drawing on evidence supporting its impact, we can address the unmet needs of this vulnerable population.

## Data Availability

Data sharing is not applicable to this article as no datasets were generated or analyzed during the current study.

## Abbreviations

HAE: Hereditary angioedema
HTC: Hemophilia Treatment Center
IV: Intravenous
ED: Emergency department
CHAEN: Canadian Hereditary Angioedema Network
